# Soil Moisture Sensing via Swept Frequency Based Microwave Sensors

**DOI:** 10.3390/s120100753

**Published:** 2012-01-11

**Authors:** Mathew G. Pelletier, Sundar Karthikeyan, Timothy R. Green, Robert C. Schwartz, John D. Wanjura, Greg A. Holt

**Affiliations:** 1 USDA-ARS, Cotton Production and Processing Unit, Lubbock, TX 79403, USA; E-Mails: john.wanjura@ars.usda.gov (J.D.W.); greg.holt@ars.usda.gov (G.A.H.); 2 Rain Bird Corp., San Diego, CA 92123, USA; E-Mail: skarthikeyan@rainbird.com; 3 USDA-ARS, Agricultural Systems Research Unit, Fort Collins, CO 80526, USA; E-Mail: tim.green@ars.usda.gov; 4 USDA-ARS, Soil and Water Management Research Unit, Bushland, TX 79012, USA; E-Mail: robert.schwartz@ars.usda.gov

**Keywords:** TDR, cotton moisture, moisture sensing, permittivity, microwave sensing, microwave moisture, saline, salinity

## Abstract

There is a need for low-cost, high-accuracy measurement of water content in various materials. This study assesses the performance of a new microwave swept frequency domain instrument (SFI) that has promise to provide a low-cost, high-accuracy alternative to the traditional and more expensive time domain reflectometry (TDR). The technique obtains permittivity measurements of soils in the frequency domain utilizing a through transmission configuration, transmissometry, which provides a frequency domain transmissometry measurement (FDT). The measurement is comparable to time domain transmissometry (TDT) with the added advantage of also being able to separately quantify the real and imaginary portions of the complex permittivity so that the measured bulk permittivity is more accurate that the measurement TDR provides where the apparent permittivity is impacted by the signal loss, which can be significant in heavier soils. The experimental SFI was compared with a high-end 12 GHz TDR/TDT system across a range of soils at varying soil water contents and densities. As propagation delay is the fundamental measurement of interest to the well-established TDR or TDT technique; the first set of tests utilized precision propagation delay lines to test the accuracy of the SFI instrument’s ability to resolve propagation delays across the expected range of delays that a soil probe would present when subjected to the expected range of soil types and soil moisture typical to an agronomic cropping system. The results of the precision-delay line testing suggests the instrument is capable of predicting propagation delays with a RMSE of +/−105 ps across the range of delays ranging from 0 to 12,000 ps with a coefficient of determination of r^2^ = 0.998. The second phase of tests noted the rich history of TDR for prediction of soil moisture and leveraged this history by utilizing TDT measured with a high-end Hewlett Packard TDR/TDT instrument to directly benchmark the SFI instrument over a range of soil types, at varying levels of moisture. This testing protocol was developed to provide the best possible comparison between SFI to TDT than would otherwise be possible by using soil moisture as the bench mark, due to variations in soil density between soil water content levels which are known to impact the calibration between TDR’s estimate of soil water content from the measured propagation delay which is converted to an apparent permittivity measurement. This experimental decision, to compare propagation delay of TDT to FDT, effectively removes the errors due to variations in packing density from the evaluation and provides a direct comparison between the SFI instrument and the time domain technique of TDT. The tests utilized three soils (a sand, an Acuff loam and an Olton clay-loam) that were packed to varying bulk densities and prepared to provide a range of water contents and electrical conductivities by which to compare the performance of the SFI technology to TDT measurements of propagation delay. For each sample tested, the SFI instrument and the TDT both performed the measurements on the exact same probe, thereby both instruments were measuring the exact same soil/soil-probe response to ensure the most accurate means to compare the SFI instrument to a high-end TDT instrument. Test results provided an estimated instrumental accuracy for the SFI of +/−0.98% of full scale, RMSE basis, for the precision delay lines and +/−1.32% when the SFI was evaluated on loam and clay loam soils, in comparison to TDT as the bench-mark. Results from both experiments provide evidence that the low-cost SFI approach is a viable alternative to conventional TDR/TDT for high accuracy applications.

## 1. Introduction

Accurate measurement of moisture content is a key requirement in hydrological, geophysical and bio-geochemical research as well as for material characterization, process control and irrigation efficiency in water limited regions. Within these areas, consideration of the surface area and associated bound water content [1] is becoming increasingly important for providing answers to many fundamental questions ranging from characterization of cotton fiber maturity, to accurate dielectric measurement methods of soil water content for hydrological assessment and efficient irrigation practices. One promising technique to address the increasing demands for higher accuracy water content measurements is the utilization of electrical permittivity characterization of materials as a proxy for water content. This proxy has enjoyed a strong following in the soil-science and geological community through measurements of apparent bulk permittivity via time-domain-reflectometry (TDR) as well in many process control applications. However, many current applications require accuracies beyond that available from traditional TDR and would benefit by removal of the inherent difficulties associated with TDR’s requisite waveform interpretation. The most logical pathway to enhanced accuracy lies in a transition from time-domain based TDR measurements towards a frequency-domain based network analyzer style measurement of the bulk complex permittivity that will allow for removal of the adverse effects that high surface area soils and electrical-conductivity, due to elevated soil-salinity, imparts onto the measurements of apparent bulk permittivity that is utilized in the traditional TDR approach. Unfortunately, network analyzer style measurements, while known for their accuracy, are an expensive alternative which typically precludes its use except for the most demanding research applications. Thus a need exists for a low-cost high-accuracy frequency domain based measurement approach. This study assesses the performance of a new microwave swept frequency domain based instrument (SFI) that has promise to provide a lower-cost high-accuracy alternative to the traditional and more expensive TDR with the inherent advantages afforded by the frequency domain net-analyzer approach which has potential to lead to higher accuracy solutions than available through traditional TDR systems.

Frequency domain analysis of soils, cotton lint, biological cells and media is rapidly gaining appreciation due to the ability to provide a true measurement of bulk complex permittivity, as opposed to an apparent bulk-permittivity measurement, with its inherent low loss assumption, that TDR analysis in the time domain provides. The advantage of the frequency domain approach, above 500 MHz, is the ability to handle a significant reduction in signal strength as well as the signal attenuation’s influence on the real portion of the dielectric permittivity, TDR’s primary measurement, caused by soil salinity and dielectric damping from high-surface area soils which in turn lead to degradation for the estimation of the bulk permittivity as a result of frequency dependent attenuation [1,2]. Of particular concern, in the correction of bulk permittivity for losses due to salinity in TDR, is the currently accepted use of the low frequency signal attenuation, as a surrogate for direct-current, DC, conductivity measurement which in turn is used as a proxy to estimate a correction factor for the high frequency dielectric loss in the bulk permittivity. Unfortunately, in current conventional practice [2] this step effectively utilizes the attenuation estimate from the 10 kHz TDR/TDT square-wave signal to estimate dielectric loss at the effective bandwidth of TDR which is typically 100–300 MHz for the wet soils that benefit from this correction (dry soils are typically low-loss and are unaffected so this step is unnecessary). As the attenuation correction factor is not available from the TDR analysis at the frequency of interest, the use of the proxy for the DC conductivity term is used for correction of the apparent bulk permittivity to that of real portion of the complex permittivity, which is still very much an experimental effort [1]. Of particular note is that the need for the DC proxy can be avoided if one performs the measurement in the frequency domain as then the attenuation is directly measured at the frequency of interest. Mathematically it can be shown that both the DC conductivity term as well as the dielectric loss at the frequency of measurement both impacts the obtained permittivity measurement by looking at the propagation coefficient for a plane wave propagating in a source free environment [3], Equations (1–4):

$$\gamma =\mathrm{jk}=\alpha +j\beta =j\sqrt{\mu \varpi^{2}\varepsilon \prime \left(1-j\left(\frac{\varpi \varepsilon \prime \prime +\sigma }{\varpi \varepsilon \prime }\right)\right)}=j\varpi \sqrt{\mu \varepsilon \prime }\sqrt{\left(1-j\left(\frac{\varpi \varepsilon \prime \prime +\sigma }{\varpi \varepsilon \prime }\right)\right)}$$

then letting:

$$\left(\frac{\varpi \varepsilon \prime \prime +\sigma }{\varpi \varepsilon \prime }\right)=\text{tan}\;\delta$$

provides:

(1) $$\gamma =\alpha +j\beta =j\varpi \sqrt{\mu \varepsilon \prime }\sqrt{\left(1-j\left(\frac{\varpi \varepsilon \prime \prime +\sigma }{\varpi \varepsilon \prime }\right)\right)}=j\varpi \sqrt{\mu \varepsilon \prime }\sqrt{\left(1-j\;\text{tan}\;\delta \right)}$$

where:

γ = propagation coefficient (1/m).
ε′ = real, dielectric constant, term of the complex permittivity (F/m).
ε″ = imaginary, loss, term of the complex permittivity (F/m).
α = attenuation factor of the propagation coefficient (nepers/m).
*j* = unit imaginary number √−1.
β = phase delay factor of the propagation coefficient (radians/m).
σ̣ = conductivity factor of the propagation coefficient at DC {0 Hz} (S/m).
μ̣ = material permeability (H/m).
*ϖ* = omega (radians/s).

Further noting for soil where the magnitude of (ε″/ε′) < 1, Equation (1) can be expanded via a power series to:

(2) $$\alpha +j\beta =j\varpi \sqrt{\mu_{0}\varepsilon \prime }\left(1-j\;\text{tan}\;\delta )\right)\frac{1}{2}=j\varpi \sqrt{\mu_{0}\varepsilon \prime }\left(\sum_{n=0}^{\infty }\left(\overset{\frac{1}{2}}{\underset{n}{}}\right)x^{n}\right)$$

This leads to the approximation Equations (3) and (4), which relates the frequency domain network analyzer measured attenuation and the delay term, α and β respectively, to the complex permittivity with the dc conductivity term intact:

(3) $$\alpha =\varpi \sqrt{\mu_{0}\varepsilon \prime }\left(\frac{1}{2}\left(\frac{\varpi \varepsilon \prime \prime +\sigma }{\varpi \varepsilon \prime }\right)-\frac{1}{16}{\left(\frac{\varpi \varepsilon \prime \prime +\sigma }{\varpi \varepsilon \prime }\right)}^{3}+\frac{7}{256}{\left(\frac{\varpi \varepsilon \prime \prime +\sigma }{\varpi \varepsilon \prime }\right)}^{5}\right)$$

(4) $$\beta =\varpi \sqrt{\mu_{0}\varepsilon \prime }\left(1+\frac{1}{8}{\left(\frac{\varpi \varepsilon \prime \prime +\sigma }{\varpi \varepsilon \prime }\right)}^{2}-\frac{5}{128}{\left(\frac{\varpi \varepsilon \prime \prime +\sigma }{\varpi \varepsilon \prime }\right)}^{4}+\frac{21}{1024}{\left(\frac{\varpi \varepsilon \prime \prime +\sigma }{\varpi \varepsilon \prime }\right)}^{6}\right)$$

For applications where the complex permittivity ratio of ε″/ε′ ≤ 0.5 (most soils); the higher order terms can be discarded with less than a 1% imparted error which leads to the simplified equations shown in Equations (5) and (6):

(5) $$\alpha =\varpi \sqrt{\mu_{0}\varepsilon \prime }\left(\frac{1}{2}\left(\frac{\varepsilon \prime \prime }{\varepsilon \prime }\right)\right)=\sqrt{\mu_{0}\varepsilon \prime }\left(\frac{1}{2}\left(\frac{\varpi \varepsilon \prime \prime +\sigma }{\varepsilon \prime }\right)\right)==\sqrt{\mu_{0}\varepsilon \prime }\left(\frac{1}{2}\left(\text{tan}\;\delta \right)\right)$$

(6) $$\beta =\varpi \sqrt{\mu_{0}\varepsilon \prime }\left(1+\frac{1}{8}{\left(\frac{\varepsilon \prime \prime }{\varepsilon \prime }\right)}^{2}\right)$$

In summary, while the approach of the researchers listed in [1] has shown positive results for correcting the attenuation impact on the TDR obtained apparent bulk permittivity towards that of a corrected real portion of the complex permittivity, it is important to recognize the benefits for high accuracy work of obtaining the measurements directly in the frequency domain as you can then avoid the use of a DC surrogate for the required frequency dependant dielectric loss term, as they are truly distinctly different and should be treated as such.

For the sake of convenience to the reader, the process of taking the propagation delay measurement, obtained from TDR/TDT, to an estimate of bulk permittivity is shown here:

$$v_{p}=\frac{c}{\sqrt{\varepsilon_{r}^{*}\;\mu_{r}}}$$

Re-arranging, and assuming non-magnetic media where μ_r_=1, leads to:

(7) $$\varepsilon_{r}^{*}=\;\varepsilon_{r}^{\prime }+\varepsilon_{r}^{\prime \prime }={\left(\frac{c}{v_{p}}\right)}^{2}$$

where:

ν*_P_* = signal propagation velocity (m/s).
ε_r_^*^ = relative complex permittivity = ε_r_’ + ε_I_’’.
c = speed of light (m/s).

Noting the complex permittivity doesn’t lend itself well in TDR analysis; typically in the industry, a low-loss assumption is made which leads to the use of propagation delay, or velocity in obtaining an estimate of the apparent bulk permittivity by dropping the dielectric loss:

(8) $$\varepsilon_{r}^{*}\;(\mathrm{low}–\mathrm{loss})=\;\varepsilon_{r}^{\prime }={\left(\frac{c}{v_{p}}\right)}^{2}$$

Use of Equation (8) however ignores the impact of dielectric losses on the signal propagation, as detailed in Equation (6). Thus, for the most demanding work there are two alternatives by which to avoid the errors associated with the low loss approximation inherent in the apparent bulk permittivity approach:

1. Obtain the measurement in the frequency domain so as to be able to apply Equations (5) and (6) directly,
  or
2. Convert the time domain signal into the frequency domain to again apply Equations (5) and (6).

For the case of the SFI instrument, the measurement obtained are described in detail in [4–7]. A few pertinent notes of distinction between the SFI technique and TDR/TDT are:

- SFI provides a difference frequency that is directly proportional to the signal’s propagation time delay, thereby providing an equivalent measurement to TDR/TDT, with the exception that, unlike TDR, the frequency is controlled the frequency band, and bandwidth, can be selected a to obtain the signal velocity at any desired frequency.
- The attenuation coefficient can also be obtained, directly at the frequency of interest.
- As the frequency is controlled and is deterministic, the full bulk complex permittivity can be obtained for each interrogation band yielding network analyzer equivalent measurements. This in turn allows for the use of the extra degree of freedom provided by multi-band permittivity estimations that can be then used to sense the soil type and provide an auto-calibration routine the sensor, something that is not currently achievable with TDR.

**Figure 1.**
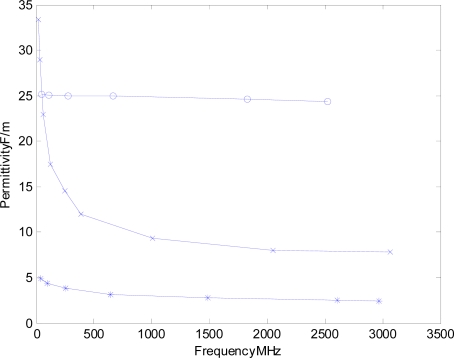
Illustration detailing the dramatic differences in the permittivity response of sand “o” near saturation, versus bentonite clay “x” at 30% volumetric moisture, and bentonite clay “*” at 5% volumetric moisture (m/m). Of particular note is the additional information available in the frequency domain that is available, from a wide-bandwidth interrogation of the soil, which has the potential for the development of auto-calibration algorithms which can sense the soil type, without the need for a laboratory calibration which is currently required in high accuracy field work.

Noting that both the network analyzer technique and the SFI instrument, we are investigating herein, obtain their measurements in the frequency domain. Thus, measurements obtained with either system can utilize Equations (5) and (6) to directly obtain the real portion of the bulk complex permittivity and remove the error provided by dielectric damping, that the low-loss apparent permittivity approach of TDR/TDT cannot resolve. Of particular concern occurs in heavier soils where the relaxation of the permittivity causes an added delay to the obtained permittivity, Equations (5) and (6) and Figure 1 [1,3]. While we note that there has been some work in utilizing the DC conductivity as a proxy, or the 10kHz decayed amplitude in TDR, as can be appreciated, for soils with varying levels of soil salinity, this proxy has significant limitations in practice. Thus, the network analyzer approach and the SFI technique both provide significant advantages over TDR/TDT as utilized in the normal operational configuration.

The alternative and more recent approach for TDR/TDT that seeks to alleviate these disadvantages takes the approach #2, discussed in the previous paragraph, which seeks to convert a digitized time domain signal into the frequency domain, for extraction of the signal’s magnitude, or attenuation coefficient “α”, and phase delay, “β”, at the frequency/s of interest via a Fourier Transform. While this approach in theory would allow for derivation of the true complex bulk permittivity, as opposed to the low-loss apparent permittivity approximation [2,8–11], in practice there are complications buried in the details of implementation. And while it is noted in the referenced articles, as well as by many that are familiar with the art, the equivalence, via a Fourier Transform, “FT”, of time domain to frequency domain conversions; what is not typically appreciated is that in TDR, the system utilizes a step input, rather than an impulse interrogation signal. And it is the use of the step input which imparts the detrimental impact on the application of the Fourier transformation. The degradation being due to the fact that the step input has the equivalent operation in the frequency domain, as an integration operation or low-pass filter. Thus, in order to obtain the actual impulse response, the analysis has to undo this integration operation, caused by the step input, by means of a digital approximation to a derivative which is frequency dependant depending upon how it’s implemented. This class of problems is well known in the digital signal process literature as the class of problems known as de-convolution solutions [12].

While the advantages of a frequency domain approach has been recognized and reported by other researchers [13,14]. There are still further advantages that have yet to be explored that can be achieved by utilizing a multi-spectral approach where the potential can be seen by noting the dramatic differences in frequency spectrum of the real term of the soil’s permittivity in the comparison between low surface area soils, such as sand, in comparison to high surface area soils rich in clays, Figure 1.

As can be readily appreciated in the permittivity response, as a function of frequency, high clay content soils greatly depress the permittivity in the higher frequency bands (>300 MHz) resulting in a profound impact on the measured apparent bulk permittivity measured by TDR which can lead to large errors and magnify temperature dependencies as the measurement degrades to one that becomes frequency dependant, as well as soil-moisture and density dependant due to the frequency dependant variation in filtering provided by the soil probe interactions with the varying loss and delay properties as the soil changes from wet to dry. These effects could be almost entirely removed if an accurate measurement was obtained at multiple frequencies as each frequency measurement occurs at a known and fixed frequency and can then be leveraged via multivariate spectral analysis such as the commonly used principal-component-analysis, “PCR” and partial-least-squares, “PLS”, techniques that are used in optical and near-infrared spectroscopy. This extended ability, provided by the frequency based approach, is especially valuable for materials that provide relaxing responses where the permittivity changes with frequency [15]. However one of the major impediments to rapid adoption of this approach is the significant economic costs associated with applying frequency domain network analyzer type measurements under field conditions.

In an effort to provide a lower cost, and hence more accessible, frequency domain technology; the focus of this research examines a similar approach that utilizes a direct measurement in the frequency domain utilizing a hybrid technique that is based on a recently developed swept frequency technology [4–6]. In this research effort, the swept frequency approach was further refined with the addition of a low cost electronic calibration system with the further improvement provided by the addition of an ultra-wideband spectral foot-print that provides enhanced accuracy of the obtained measurements of propagation delay which can be readily converted to permittivity as needed [7].

The experimental objective of this research was to characterize the accuracy of an experimental SFI, based upon USDA-ARS *US Patent 7,135,871,* as applied for use in volumetric soil water content measurements. This research, reported herein, utilized a high-end Hewlett Packard^1^, “HP”, 12 GHz time-domain oscilloscope for comparison with dielectric measurements of the low-cost experimental SFI moisture-sensing technology. The use a higher frequency TDR/TDT, than the typical 2 GHz Tektronic cable tester, was necessary as the research is interested in the potential for utilizing shorter probes, 16.5 cm, as attenuation loses with TDR probes in heavy clay soils are known to lose the return pulse, especially at elevated temperatures. This shorter probe in turn requires the need and use of higher frequencies to provide the required accuracy, especially with dry soils. Additional benefits to the use of higher accuracy HP TDR/TDR instrument is seen as the SFI instrument can be configured to operate across an extremely wide frequency band that can span from 300 MHz to over 3.0 GHz. We also note that while the exceptionally high bandwidth of the HP instrument was not required for this research, the extra head-room in bandwidth afforded by the use of the HP TDR/TDT provides for the highest possible confidence in comparison between the HP TDT and the SFI instrument. Additionally because the through transmission measurements provides a significant reduction in the impact of impedance miss-match on the measurement, all measurements were performed in a through-transmission (TDT) configuration instead of the more traditional reflection topology (TDR) which provides another benefit towards utilizing an HP TDR/TDT instrument, as the typical Tektronix cable tester is only configured for a TDR measurement and cannot readily perform a TDT measurement. We do however note that due to the need for an minimally invasive soil probe, that our laboratory is currently working on the development of a practical insertion TDT probe for use with our SFI technology as the argument has been broached that TDT is not a practical solution due to the lack of an insertion probe. The experiment that is the focus of this paper was designed to provide a 1:1 comparison, between the HP TDT instrument’s responses, to that of the experimental SFI swept frequency response, as the key experimental measure of performance rather than utilizing the more traditional comparison for prediction of moisture content, which will be left to future research efforts. As the details of the implementation of an SFI instrument has been well documented in the literature by the authors, the reader is referred to the references cited herein for the specifics on this approach [4–7,16].

## 2. Methods and Procedures

In characterizing the experimental swept frequency system, a two-part approach was designed to first characterize the system’s accuracy independently of a soil-sensing structure and then later in combination with a high quality soil-sensing structure that was designed to minimize the influence of the sensing structure while still subjecting the experimental SFI sensing technology to a range of soil types ranging from sand to loam to clay-loam.

In characterizing an instrument such as TDR, or TDT, that provides a measurement of propagation delay that is then converted mathematically to a measurement of bulk apparent permittivity, the most accurate means to characterize the instrumentation independently of the soil-sensing-probe interaction is to test the instrument against high-precision delay lines, thereby removing from consideration the influence of the soil, and the soil to soil-probe interaction, in the evaluation of the instruments ability to accurately measure propagation time-delay caused by the changing media’s electrical properties. This proposed technique, utilized in the first phase of this research, is designed to isolate the potential performance of the instrument from the rigors and demands of all the issues associated with interfacing a sensor to soil with the primary benefit being that an independent delay-line test provides insight into the potential accuracies that could be achievable given an ideal soil-sensing-probe design.

Of particular note of interest, in the selection of an instrument only test protocol, was the potential use of permittivity standards by which to characterize the accuracy of the system. While we concur that when also using a probe, permittivity standards [15] provide a grounding basis by which to ascertain that a system is achieving reasonable answers. However, for characterizing an unknown instrument for accuracy, permittivity standards provide too few options and permittivity values unless one starts mixing standards together and then the precision of an absolute permittivity standard is significantly compromised, even though it is a simple task to quantify the percentage of the mixture through wet chemical analysis, as one is still left with the problem of how to estimate the permittivity of the mixture through the use of multi-term Debye or Cole-Cole functions. Further given that in today’s TDR systems, propagation delay is the primary measurement of interest as it is well known to have acceptable measurement accuracies when applied to volumetric soil-moisture measurements. Thus, propagation delay standards via precision delay lines offers the following significant advantages over utilizing dielectric permittivity standards comprised of solutes and solutions, as delay line propagation standards provide:

- 50 Ω lines, thus impedance miss-match issues and corrections are eliminated.
- Precision delay lines are well characterized from the manufacturer and can easily be tested on both network analyzers and TDT systems for accuracy which provides another quality control step in the comparison between an experimental unit to that of high quality TDT system.
- A wide range of delays and delay combinations are easily created and thereby can provide exhaustive testing samples, by which to run an experimental unit through a bed of nails testing protocol that is extremely difficult if not impossible to achieve, when utilizing the more traditional permittivity standards based on oils, alcohols and other solutes and solutions.
- As frequency domain sensors typically have less accuracies when either the reference reading or the sample are at or near +/−180°, it is important to not only test a given propagation delay but also to test this same delay across a range of sensor locations within the 0–360° span. Again, propagation delay lines provide an ideal platform for this analysis.
- A propagation delay line allows for the removal of the interfacing probe from the assessment and thereby provides insight into the accuracy of the sensing electronics, independently of other issues associated with impedance miss-match due to soil-probe designs coupled with the wide range of permittivity that soil imparts to the sensor as it swings from dry to saturated. As such, delay lines can provide insight into the design and can be utilized to rapidly ascertain important issues such as where improvements can be made or alternatively where they can be relaxed, and still meet the design specification, so as to achieve a lower cost design. In summary they are a critical tool in the toolbox for sensor development.

Given the significant advantages of propagation delay lines versus permittivity standards, as discussed in the previous paragraph, the first phase of the research utilized a series of precision delay lines that were measured for propagation delay via a high-end 6 GHz HP frequency domain network analyzer, as a high quality network analyzer provides one of the most accurate measurements known for characterizing delay lines. To ensure the phase ambiguity of the network analyzer measurement was correctly resolved, all delay lines were also measured on an HP 12 GHz TDT to ensure the full unwrapped phase delay was correctly quantified by the network analyzer and was then further compared to the precision delay line manufacturer’s specification whereby all three, network analyzer, TDT and manufacturer’s specification, were found to be in good agreement. This ensured the research a high accuracy delay line standard by which to judge the SFI technique’s response to each delay line. The testing protocol replicated each delay line measurement with varying lengths of lead-in cables, in order to alter the specific phase at which the measurement was obtained, so as to ensure a stable and repeatable measurement that was exercised across the complete range of possible starting and stopping phase positions. It should be further noted that as the SFI technique also provides a direct measure of propagation delay, no calibration was performed on the instrument for the delay line test. All comparisons were performed based upon first principles which effectively ties the measurement directly back to Maxwell’s propagation equations for electromagnetic waves [4–7,17].

**Figure 2.**
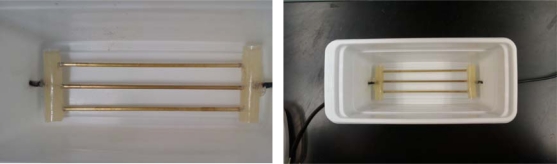
Experimental metal-tined TDT probe, that provided the basic interface between the experimental swept frequency system under test, and the TDT validation instrument. Each metal tine is nominally 165 mm long.

The sensing structure developed for part two of this testing was a short 16.5 cm tri-probe through-transmission line configuration designed to propagate transverse-electromagnetic (TEM) waves from low MHz through to the low GHz microwave region (Figure 2). Network analyzer testing of the soil probe, characterized in air, was found to have a bandwidth range from 0 to just over 2.5 GHz.

### Experimental Protocol

The system was tested in three soils: sand, with a 6.5% silt fraction, and two local soils, an Olton clay loam, obtained from the A horizon in the taxonomic class of a fine mixed superactive thermic aridic paleustolls and an Acuff loam also obtained from the A horizon that is a fine-loamy mixed superactive thermic aridic paleustolls. Each soil was procured from the field, air dried, and split into multiple lots with each lot being brought to a different level of volumetric soil water content so as to achieve 10 levels of saturation percentages by which to evaluate the sensor response. The air dry soils original gravimetric volumetric water content was measured and then the requisite volume of water was added to each sample lot to bring its moisture up to the target moisture content level. Each soil was divided into 500 mL lots and water was added to each lot to bring the moisture content to one of the 10 target water content levels ranging from air dry to field capacity. Of particular note was the atypically high water holding capacity of the selected sand, which is most likely due to the substantial silt fraction which effectively raised the total water holding capacity. In order to avoid free water in the sample from being lost and biasing the test, the final target moisture for this experiment was limited to at or below field capacity by rejecting any samples exhibiting free water in the sample storage container after sample had obtained equilibrium. After adding water to each lot to bring the lot to the target volumetric water content, the soil was thoroughly mixed and then allowed to equilibrate for a minimum of 5 days.

In an effort to provide the highest-quality interface between the instruments and the circuitry under test, all tests utilized a non-insulated 16.5 cm metal-tined through-transmission probe constructed out of brass rods that were directly soldered to the interfacing coaxial cable and then embedded in epoxy to obtain a water-tight sensing platform. The experimental TDT probe was then placed inside a plastic housing, with a water tight seal, to allow for testing of liquids and saturated soil-pastes (Figure 2).

The testing protocol required filling the test chamber with one of the prepared sand/soil lots, at one of the target volumetric water content levels, at one of three bulk densities (loose, lightly packed, packed). While we note that bulk density is a significant factor in the electromagnetic response, for a given moisture content, quantitative measures of bulk density were designed out of the protocol due to the difficulty in packing the experimental soil cells to a uniform density across the full range of moisture contents. This protocol was repeated for each of the moisture contents provided by the lots, yielding a total of 30 readings for each of the soils on the experimental SFI instrument that was being evaluated, as well as on the high-end HP 12 GHz TDT instrument, where the display was configured for 500 ps/division with a 60 ps rise time on the interrogation 50 Ω step-response signal that the TDT instrument supplied. For each soil sample preparation, the material was placed into the sample chamber and then shaken to provide the first soil density of the “loose” category. The metal-tined TDT-probe, inside the soil-packed test chamber, was then connected to the TDT-instrument to obtain the high-quality estimate of propagation delay after which the TDT-probe was subsequently connected to the experimental SFI, without disturbing the sample, to obtain the experimental SFI instrument’s comparison reading for the same sample as presented to the TDT-probe. In the above outlined protocol, the experimental protocol was designed to provide a near identical propagation delay to each instrument for the soil/TDT probe combination under-test with the only deviation being to perhaps some limited sample shifting during the coaxial cable relocation between the two instruments. Thereby the experimental protocol was designed to subject each instrument to the exact same soil-type/soil-moisture/density/soil-probe so that each system was measuring identical effective bulk permittivity’s, as provided by the test sample. In order to also allow for examination of the impact of salinity, a saturated paste sample at an elevated solution conductivity of 6 dS m^−1^ was also added to the test, one for the Acuff loam and one for the Olton clay loam, that was achieved by leaching a 6 dS m^−1^ solution through the soil sample until the soil salinity had stabilized at 6 dS m^−1^.

Of particular note herein for the objectives of this research, the moisture content range and the response of the experimental sensor in comparison to the high-end HP 12 GHz TDT response was the primary consideration under evaluation. Thus the moisture contents were only used as a means to evaluate the influence of dielectric relaxation and conductivity on the sensors response across a range of soil water contents. This modified protocol was a response to the lack of precision in the repeatability that the packing of the soil into the test chamber creates as the volumetric soil moisture changes. Thus, for the purposes of this research, the fundamental criteria under investigation was the ability of the experimental sensor to track the TDT’s system response as it provides a solid basis by which to evaluate the suitability of the SFI instrument for use in soil moisture sensing.

## 3. Results and Discussion

**Figure 3.**
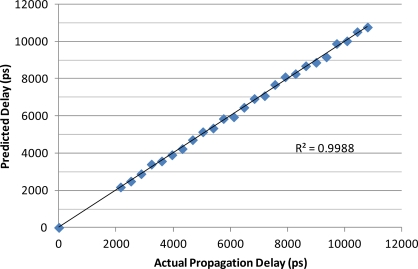
Propagation delay comparison between precision delay lines and the experimental Swept Frequency System (regression line shown).

The results of the precision delay line testing of the SFI instrument are shown in Figure 3 and resulted in a root-mean-squared error (RMSE) of 105 ps for propagation delay across the span of 0 to 12,000 ps of delay, with a coefficient of determination of r^2^ = 0.998 and a bias of 7.24 ps.

**Figure 4.**
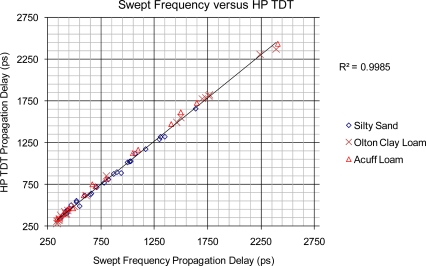
Propagation Delay (ps) comparison between a high-end TDT, 12GHz bandwidth, and the experimental Swept Frequency System for sand, Olton clay loam and Acuff loam.

The primary correlation of interest in the second phase of the research was to characterize the low-cost SFI instrument’s ability to measure the propagation delay that a given soil sample provides when interrogated by the instrument via a soil-packed metal-tined TDT-probe, at varying levels of volumetric water content across a range of bulk densities. The results from the testing are detailed in Figure 4 and show a high correlation between the predicted propagation delays, provided by the SFI instrument in comparison to the standard method for propagation delay measurements provided by the high-end 12 GHz TDT instrument. The calibration coefficient linking the TDT-probe to propagation delay for the SFI instrument was developed on the sand and was then used to predict the delay response on the Olton clay loam and Acuff loam, thereby providing a verification characterization of the SFI instrument’s ability to track a calibration over an independent set of soils. Over the verification samples (Olton and Acuff), the SFI instrument responded with a mean bias of 26.4 ps with a root-mean-square-error, “RMSE” of 31.7 ps when compared to the measurements performed with the high-end HP TDT instrument, Figure 4.

## 4. Conclusions

The initial phase of the study examined the experimental SFI instrument against precision delay lines and found the instrument to provide an RMSE of calibration at +/−105 ps with a bias of 7.24 ps. As the full span for this study was across the span of 12,000 ps, this represents an instrumental accuracy of +/−0.98% based on full span, RMSE basis. Noting that the propagation delay error is fixed, the span is a function of the length of the soil probe that is being used with the instrument as well as the type of soil. Thus, these estimates only provide guidance into expected results. However, as typical soil water content sensors utilizing TDR struggle to get within 2–4%, this level of accuracy is encouraging as an alternative and lower cost option for use in high-accuracy applications.

The performance of the direct comparison performed in this study between the high-end TDT to the SFI instrument’s response over a range of soil water contents, provided a high correlation in delay times with a coefficient of determination of r^2^ = 0.998 and RMSE of 31.7 ps, when the calibration obtained on the sand was used to predict the delays provided by the Acuff Loam and the Olton Clay Loam as varied from air dry to near field capacity. This level of predictive accuracy suggests the SFI instrument is predicting the high-end HP TDT instrument at the 1.3% accuracy level. Of particular note is that the RMSE between measurements provided by the two instruments was much smaller than the typical errors reported in TDR research, which supports the widely held theory that the largest errors in TDR are most likely to be due to the “soil to soil-probe” confounding effects caused by air pockets due to soil-expansion/contraction cycles, worms and other effects, such as variations in soil-type, soil-density, and non-uniformity of volumetric soil-moisture. The positive results from this study suggest the microwave based SFI approach is a viable low-cost alternative to expensive TDR instruments and warrants further study. Based on these findings, further research is planned to evaluate the SFI technology for the potential of improving upon the accuracy of TDR instruments for use in the prediction of soil water content, by means of extraction of frequency dependant relaxation information that is readily available to frequency domain based sensors such as network analyzers and the new experimental SFI technology that is the focus of the research reported on herein.
